# Supplementary figures and images for: Evolutionary conservation of centriole rotational asymmetry in the human centrosome
Source: eLife. 2022 Mar 23;11:e72382. doi: 10.7554/eLife.72382 (PMC8983040; doi:10.7554/eLife.72382)

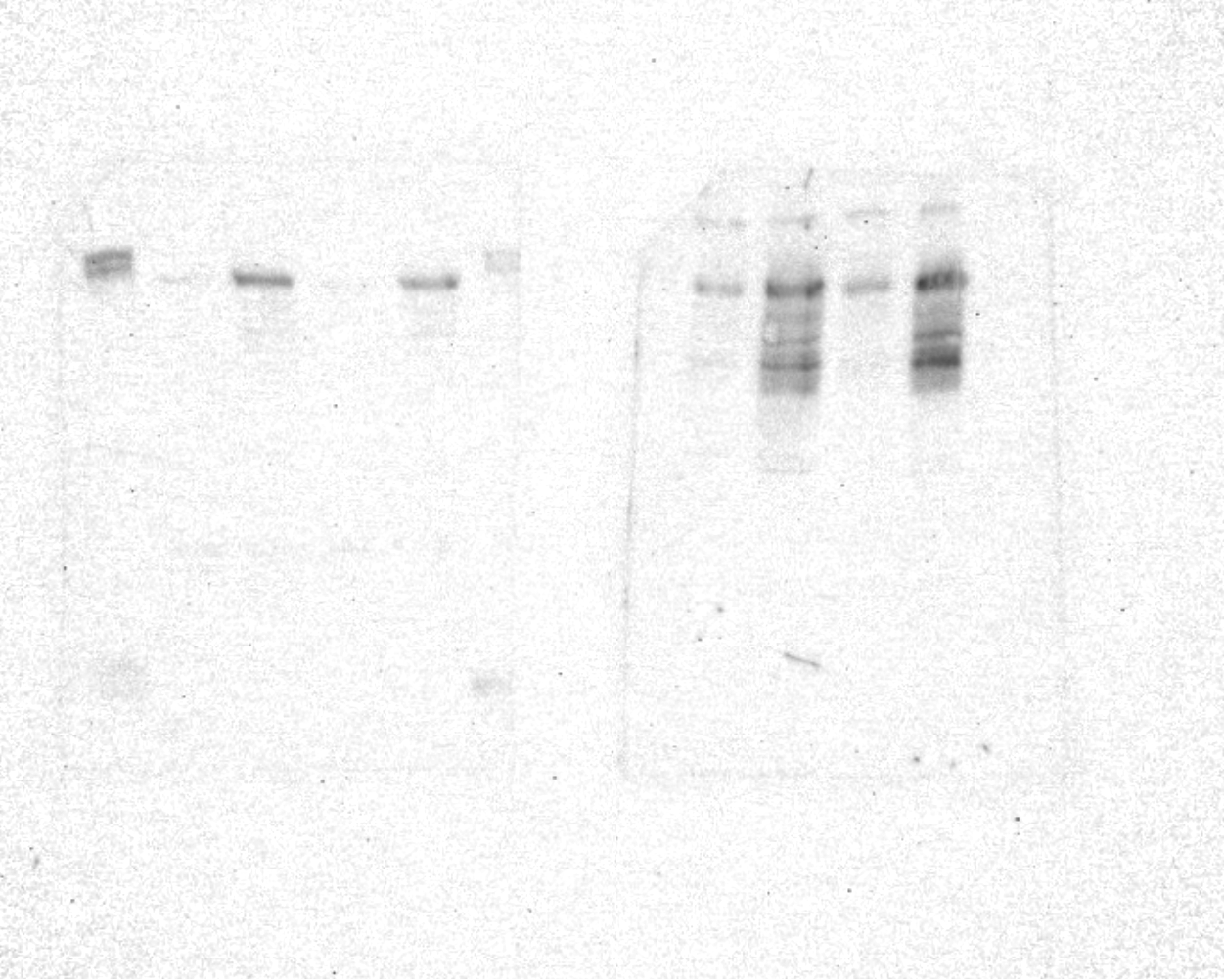

Supplement: Figure 1—figure supplement 1—source data 1. [file elife-72382-fig1-figsupp1-data1.zip › Figure1-figure supplement1-source data file 1/Figure1-figure_supplement1-source-file.tif]

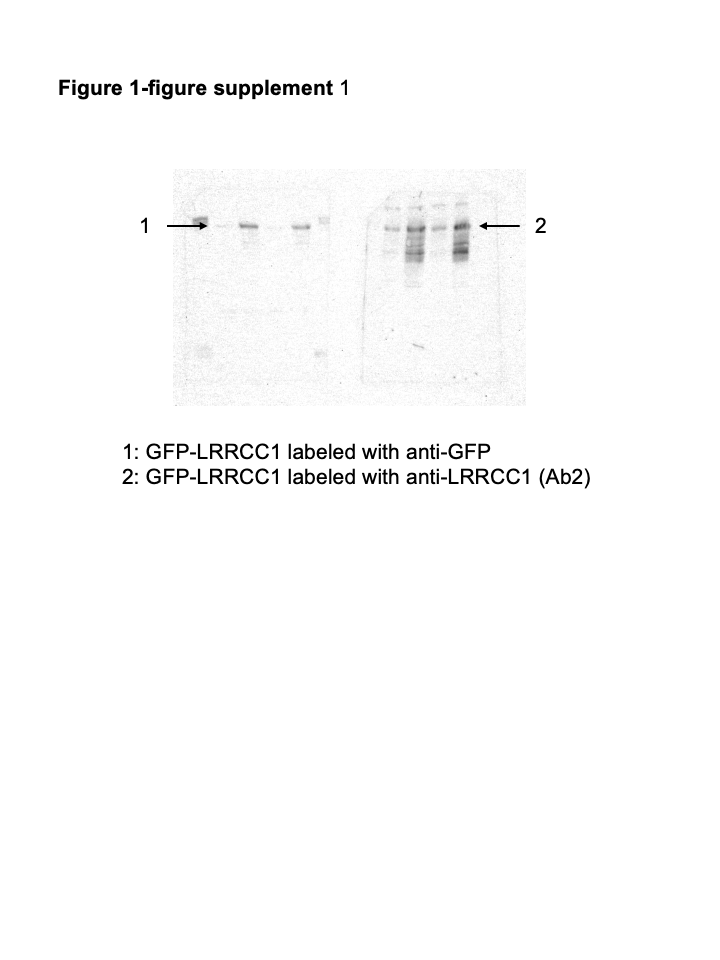

Supplement: Figure 1—figure supplement 1—source data 1. [file elife-72382-fig1-figsupp1-data1.zip › Figure1-figure supplement1-source data file 1/Figure1-figure_supplement1-source-file1.tiff]

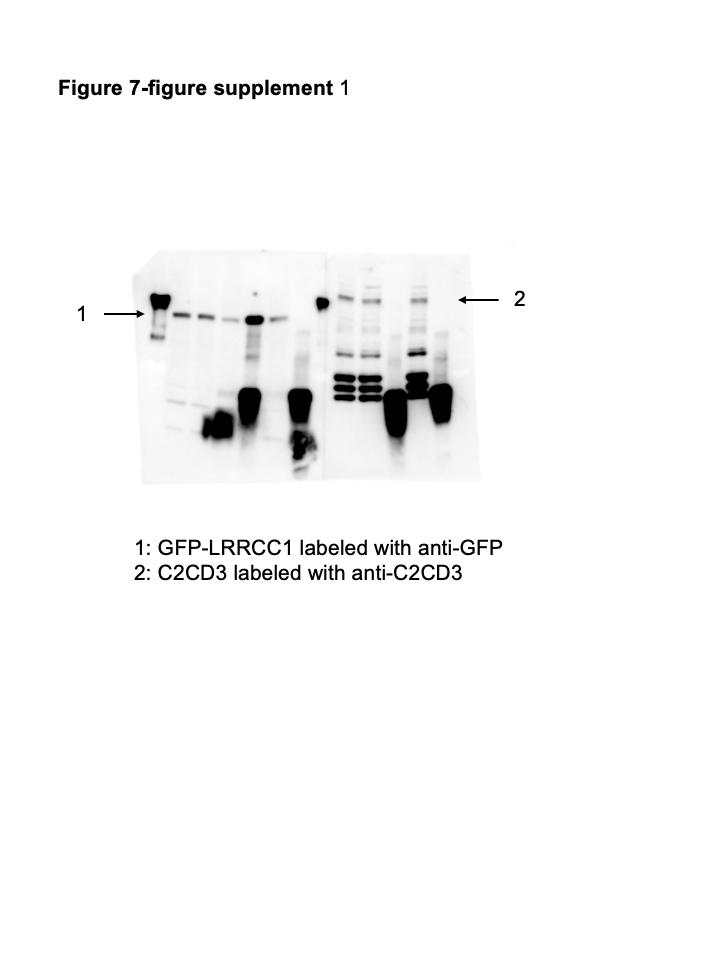

Supplement: Figure 7—figure supplement 1—source data 1. [file elife-72382-fig7-figsupp1-data1.zip › Figure7-figure supplement1-source data file 1/Figure7-figure_supplement1-source-file.tiff]
